# Outcomes of early social experiences on glucocorticoid and endocannabinoid systems in the prefrontal cortex of male and female adolescent rats

**DOI:** 10.3389/fncel.2023.1270195

**Published:** 2023-12-20

**Authors:** Laura Rullo, Loredana Maria Losapio, Camilla Morosini, Francesca Mottarlini, Sara Schiavi, Valeria Buzzelli, Fabrizio Ascone, Roberto Ciccocioppo, Liana Fattore, Lucia Caffino, Fabio Fumagalli, Patrizia Romualdi, Viviana Trezza, Sanzio Candeletti

**Affiliations:** ^1^Department of Pharmacy and Biotechnology, Alma Mater Studiorum - University of Bologna, Bologna, Italy; ^2^Department of Pharmacological and Biomolecular Sciences “Rodolfo Paoletti,” Università degli Studi di Milano, Milan, Italy; ^3^Section of Biomedical Sciences and Technologies, Department of Science, Roma Tre University, Rome, Italy; ^4^School of Pharmacy, Center for Neuroscience, Pharmacology Unit, University of Camerino, Camerino, Italy; ^5^CNR Institute of Neuroscience-Cagliari, National Research Council, Cagliari, Italy

**Keywords:** early social isolation, endocannabinoids, HPA axis, adolescence, communal nesting

## Abstract

Social and emotional experiences differently shape individual’s neurodevelopment inducing substantial changes in neurobiological substrates and behavior, particularly when they occur early in life. In this scenario, the present study was aimed at (i) investigating the impact of early social environments on emotional reactivity of adolescent male and female rats and (ii) uncovering the underlying molecular features, focusing on the cortical endocannabinoid (eCB) and glucocorticoid systems. To this aim, we applied a protocol of environmental manipulation based on early postnatal socially enriched or impoverished conditions. Social enrichment was realized through communal nesting (CN). Conversely, an early social isolation (ESI) protocol was applied (post-natal days 14–21) to mimic an adverse early social environment. The two forms of social manipulation resulted in specific behavioral and molecular outcomes in both male and female rat offspring. Despite the combination of CN and ESI did not affect emotional reactivity in both sexes, the molecular results reveal that the preventive exposure to CN differently altered mRNA and protein expression of the main components of the glucocorticoid and eCB systems in male and female rats. In particular, adolescent females exposed to the combination of CN and ESI showed increased corticosterone levels, unaltered genomic glucocorticoid receptor, reduced cannabinoid receptor type-1 and fatty acid amide hydrolase protein levels, suggesting that the CN condition evokes different reorganization of these systems in males and females.

## 1 Introduction

Early social and emotional experiences influence the neurodevelopment of individuals by affecting neurochemical substrates and behavior. Evidence exists that adequate social stimuli during the early stages of post-natal life are crucial for developing appropriate social, emotional, and cognitive skills while adverse social experiences negatively affect neurobehavioral development (Cirulli et al., 2010; Lomanowska et al., 2011). Accordingly, early life experiences shape synaptic plasticity in the prefrontal cortex (PFC) and induce life-long behavioral and persistent neurobiological changes that may increase the vulnerability to psychiatric diseases later in life (Edwards et al., 2003; Gibb et al., 2007; Wright et al., 2009; Spinhoven et al., 2010; Kolb et al., 2012; Teicher and Samson, 2013).

The hypothalamic-pituitary-adrenal (HPA) axis activation, and the consequent glucocorticoid release, is the most common physiological response to stressful stimuli (e.g., maternal separation, early social isolation) (Hill and McEwen, 2010). Glucocorticoids regulate ongoing HPA axis activity through a negative feedback loop that leads to stress response termination. However, a concerted activation of other endogenous systems is required for HPA axis regulation (Hill and Tasker, 2012). In this regard, preclinical and clinical studies highlighted that endocannabinoid (eCB) signaling is also necessary for the regulation of stress responses (Hillard, 2014; Morena et al., 2016; Maldonado et al., 2020). Indeed, it has been reported that eCB signaling disruption results in an excessive HPA axis activation, increased anxiety behavior and reduced responsiveness to rewarding stimuli (Morena et al., 2016; Micale and Drago, 2018). In this frame, it has been hypothesized that endocannabinoids (eCBs) act as co-regulators of glucocorticoid function in several stress-related brain regions (Hill and McEwen, 2009; Hillard, 2014). However, the exact molecular mechanisms by which environmental factors (i.e., maternal care, social context and stress exposure) can influence the interplay between glucocorticoid and eCB systems and the emotional development of individuals are not yet completely understood (Meyer et al., 2018; Franks et al., 2020).

We therefore investigated the impact of different forms of early social environment (i.e., social enrichment or social deprivation) on emotional reactivity and the consequent molecular alterations in plasmatic corticosterone levels, glucocorticoid and eCB systems of juvenile rats [postnatal day (PND) 35]. In particular, we assessed the gene and protein expression of glucocorticoid receptor (GR) and of two main components of the eCB system, the cannabinoid receptor type-1 (CB1R) and the fatty acid amide hydrolase (FAAH) (Trezza et al., 2012; Di Marzo and Piscitelli, 2015; Lu and Mackie, 2021).

We applied a protocol of environmental manipulation based on housing rats in either social enriched or social impoverished conditions early in life, compared to standard housing conditions. Social enrichment was realized through communal nesting (CN), consisting of housing together three pregnant rats that, upon delivery, would keep their pups together and share care-giving till weaning, a condition that provides a highly stimulating social environment to the developing pup, thereby affecting depressive- and anxiety-like responses later in life (Branchi and Alleva, 2006; Branchi, 2009; Cirulli et al., 2010). Conversely, to mimic an adverse early social environment, an early social isolation (ESI) protocol was applied during PND 14-21, an age range comparable to childhood in humans (Chini and Hanganu-Opatz, 2021). The environmental manipulations used in the present study, i.e., both ESI and CN, have been reported by previous studies to elicit behavioral, emotional and physiological alterations in adolescent rats (Bratzu et al., 2023) and adult mice (Cirulli et al., 2010; Lo Iacono et al., 2015; Catale et al., 2020) of both sexes. The aim of our study was to investigate the impact of ESI and CN in the rat male and female offspring during adolescence, a critical time window for brain maturation. Given the central role of PFC in cognitive, social, and emotional process (Dixon et al., 2017), and since endocannabinoid signaling is clearly implicated in the glucocorticoid-mediated negative feedback inhibition of the HPA axis in PFC (Hill and Tasker, 2012; Peters and Naneix, 2022), molecular investigations focused on this brain area. It is worth noting that PFC undergoes sensitive maturational processes during development and, as such, it is particularly vulnerable to early life events (Peverill et al., 2019; Smith and Pollak, 2020). In addition, PFC is also crucial for sensorimotor gating, a brain function differently affected by CN and/or ESI in a sex- and age-dependent manner (Bratzu et al., 2023). Since early postnatal experiences and rearing conditions can induce sexually dimorphic changes in CB1R expression, endocannabinoids and/or corticosterone levels (Brown and Grunberg, 1995; Suárez et al., 2009, 2010; Viveros et al., 2009; Dow-Edwards et al., 2016; Ramírez-López et al., 2017; Portero-Tresserra et al., 2018; Caradonna et al., 2022; Tran et al., 2022; Walker et al., 2022) we assessed the effects of CN or ESI in both female and male offspring to detect potential sex differences. Furthermore, the potential impact of CN condition on the effects of early social isolation has been assessed.

## 2 Materials and methods

### 2.1 Animals and nesting conditions

Wistar rats (Charles River Laboratories, Italy) weighing 250 ± 15 g were mated overnight. Pregnant rats assigned to the standard housing condition (SH) group were individually housed in Macrolon cages (40 × 26 × 20 cm; l x w x h), while pregnant rats assigned to the CN condition were housed in groups of 3 in larger Macrolon cages (62 × 44 × 22 cm; l x w x h). Both experimental groups were kept under controlled conditions (temperature 20–21°C, 55–65% relative humidity and 12/12 h light cycle with lights on at 07:00 h). Food and water were available *ad libitum*. Newborn litters found up to 17:00 h were considered to be born on that day (PND 0). One pup per litter, from different litters per treatment group, was randomly used in each behavioral experiment and not re-used in subsequent behavioral experiments, in order to avoid the so-called “litter effect” (Jiménez and Zylka, 2021).

Sample size (n) was based on our previous experiments and power analysis performed with the software G*Power 3.1. In particular, the following number of animals was used for each experiment:

(1) Maternal behavior: number of dams SH = 10 and CN = 12.
(2) Isolation-induced USVs: Males: PND 5: SH = 16, CN = 12; PND 9: SH = 16, CN = 12; Females: PND 5: SH = 16, CN = 12; PND 9: SH = 16, CN = 12.
(3) EPM test: Males: SH-CTRL = 8, SH-ESI = 8, CN-CTRL = 8, CN-ESI: 8; Females: SH-CTRL = 8, SH-ESI = 7, CN-CTRL = 8, CN-ESI: 8.

Concerning the biochemical experiments, blood and tissue samples were taken from 6 animals per group from a separate batch of rats used only for molecular studies.

The sample size for each experiment is also indicated in the figure legends. The experiments were approved by the Italian Ministry of Health (authorization n. 612/2020-PR) and performed in agreement with the ARRIVE (Animals in Research: Reporting *In Vivo* Experiments) guidelines (Kilkenny et al., 2010), the guidelines of the Italian Ministry of Health (D.Lgs. 26/14) and the European Community Directive 2010/63/EU.

### 2.2 Experimental design

#### 2.2.1 Early social isolation procedure

**FIGURE 1:**
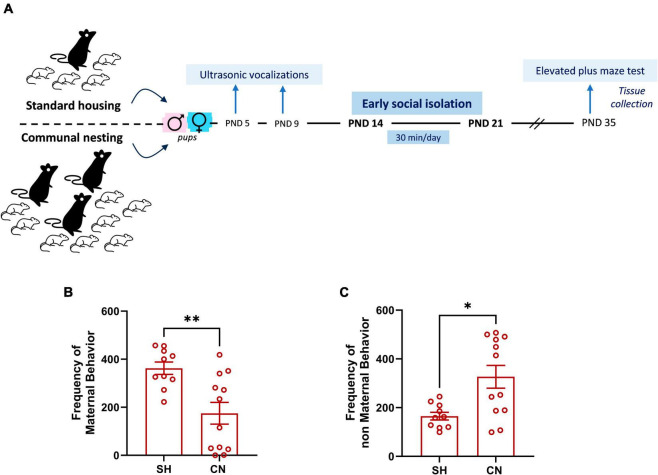
Experimental timeline **(A)** and effect of communal nesting on maternal behavior **(B,C)**. Mothers from the CN group exhibit a lower frequency of maternal behaviors **(B)** and a higher frequency of non-maternal behaviors **(C)** compared to the mothers from the SH group (SH = 10, CN = 12). Data represent mean ± S.E.M. *p < 0.05, **p < 0.01 vs. SH (Mann–Whitney test).

The protocol of environmental manipulation of rats was based on housing the animals in either social enriched or social impoverished conditions from birth to weaning (PND 0–21), compared to standard housing conditions (see timeline, Figure 1A). Social enrichment was realized through CN, an experimental procedure that consists of housing together three pregnant rats to hold their pups and share maternal care until weaning, as described above. Social deprivation consisted of a short period of social isolation (early social isolation, ESI) during the third postnatal week, an age range that in the laboratory rat is characterized by the maturation of social, sensory, motor and cognitive abilities and in which reconfigurations of neuronal structure and synaptogenesis occur in the brain (Branchi, 2009; Lister et al., 2013).

Therefore, rats were subjected to one of the following environmental conditions:

(1) Standard Housing (SH): male and female offspring born from rat dams mated individually with a male. After 1 week of mating, the female was isolated and left undisturbed until delivery. Twenty-four h after birth, the litter was reduced to 8 animals (4 males and 4 females) that were left undisturbed until weaning.
(2) Communal Nesting (CN): male and female offspring born from rat dams exposed to the CN procedure, i.e., 3 females were housed together with a male (Gracceva et al., 2009). The male was removed 1 week after mating and the 3 females were left undisturbed in the same cage until delivery. In the CN group, to approximate at naturalistic condition, deliveries were not synchronous, and pups were born within a range of 7 days in each female trio. Twenty-four h after birth, the progeny was randomly reduced to 24 animals (12 males and 12 females) that were left undisturbed until weaning.
(3) Standard Housing and Early Social Isolation (SH + ESI): male and female offspring born from rat dams mated in the SH conditions. From PND 14 to PND 21, rats were removed from the nest and singly housed in a cage with clean bedding for 30 min/day. The cage was placed on a heating pad kept at 30°C in order to prevent hypothermia.
(4) Communal Nesting and Early Social Isolation (CN + ESI): male and female offspring born from rat dams mated as in CN conditions. From PND 14 to PND 21, an ESI protocol was applied, in which each pup was singly removed from the nest and placed in a cage with clean bedding for 30 min/day.

On PND 21, pups from all experimental groups were weaned and housed in groups belonging to the same environmental condition. The behavioral experiments were carried out with female and male offspring from all experimental groups at PND 35 (adolescence).

### 2.3 Behavioral tests

#### 2.3.1 Maternal behavior observations

Maternal behavior was assessed daily in the colony room, from PND 2 to PND 13, by well-trained experimenters, blinded to experimental groups, and occurred at regular intervals of 3 min in 3 sessions of 72 min each during the light phase (09:00 a.m., 01:00 p.m., 05:00 p.m.), as previously described (Colucci et al., 2020). Literature data indicate that maternal behavior is high during the light phase and declines during the dark phase of the light/dark cycle (Champagne et al., 2003; van Hasselt et al., 2012). In fact, during the dark (active) phase, self-directed behaviors in the mothers are increased compared to pup-directed behaviors, such as licking/grooming behaviors (Champagne et al., 2003). During each session, each dam and its litter were observed every 3 min (25 observations per 3 sessions per day for a total of 75 observations per day). We measured the following 7 maternal parameters: (1) arched nursing (dam adopting a nursing posture with its back and ventral surface arched over its pups), (2) blanket nursing (dam over the pups in nursing posture but not arched), (3) passive nursing (dam adopting nursing posture lying either on its back or side), (4) licking pups (dam licking pups), (5) pup retrieval (dam moving the pups in another cage position), (6) building nest (dam manipulating nest shavings), (7) maternal self-grooming (dam grooming its breasts). We also recorded the following 4 non-maternal parameters: (1) feeding, (2) exploring (exploring the cage), (3) not-exploring without pups (dam away from the pups), (4) self-grooming (grooming its body but not the breast). The total sum of maternal and non-maternal behaviors was calculated for each litter. To investigate how maternal behavior adapts to the different needs of the pups during their development, maternal behavior was monitored from PND 2 to PND 13, i.e., a critical period during which rat pups are still developing various abilities and depend entirely on their mother for nutriment and care.

#### 2.3.2 Isolation-induced ultrasonic vocalizations

Isolation-induced ultrasonic vocalizations (USVs) are emitted by rodent pups when removed from the nest and play an important communicative role in mother–offspring interactions. The isolation-induced USVs emitted by pups from both the SH and CN groups were recorded as previously described (Melancia et al., 2018; Manduca et al., 2020) at PNDs 5 and 9 (see timeline, Figure 1A). Briefly, pups were individually removed from the nest and placed into a black Plexiglas arena (30 × 30 cm), located inside a sound-attenuating and temperature-controlled chamber. Pup USVs were detected for 3 min by an ultrasound microphone (Avisoft Bioacoustics, Berlin, Germany) sensitive to frequencies between 10 and 200 kHz and fixed at 15 cm above the arena and analyzed quantitatively (number of calls/3 min).

#### 2.3.3 Elevated plus-maze test

The elevated plus maze (EPM) apparatus comprised two open (50 × 10 × 40 cm; l × w × h) and two closed arms (50 × 10 × 40 cm; l × w × h) that extended from a common central platform (10 × 10 cm). The test was performed as previously reported (Manduca et al., 2015; D’Elia et al., 2022). At PND 35, rats were individually placed on the central platform of the maze for 5 min. Each 5-min session was recorded with a camera positioned above the apparatus for subsequent behavioral analysis carried out an observer, unaware of animal treatment, using the Observer 3.0 software (Noldus Information Technology, NL). The following parameters were analyzed:

• % time spent in the open arms (% TO): (seconds spent on the open arms of the maze/300) × 100;
• % open arm entries (% OE): (number of entries into the open arms of the maze/number of entries into open + closed arms) × 100;
• number of total arm entries: number of entries into open + closed arms.

### 2.4 Tissue and plasma collection

Another batch of rats, exposed to the same protocol described in 2.2.1, was used for molecular analysis and animals were rapidly decapitated at PND35, blood was collected, and brains were quickly removed. Brains were placed into an ice-cold plate and PFC was dissected under stereomicroscope, as a crude coronal section (from 2.0 to 4.0 mm anterior to bregma), according to the Rat Brain atlas (Paxinos and Watson, 2013). Tissues were stored at −80°C until analysis. Blood samples from each rat were collected in tubes containing EDTA (250 μL × 2 mL of blood collected) as anticoagulant agent. Plasma was separated by centrifugation (6,500 *g* for 20 min) and stored at −80°C.

### 2.5 Molecular analysis

#### 2.5.1 Analysis of plasma corticosterone levels

Corticosterone (CORT) levels were determined by an enzyme-linked immunosorbent assay (ELISA) using a commercial kit according to the manufacturers’ instructions (Tecan, Italy).

#### 2.5.2 RNA extraction and gene expression analysis by real-time qPCR

Total RNA was extracted according to the method of Chomczynski and Sacchi (2006). Each sample was subjected to DNAse treatment and converted to cDNA with the GeneAmp RNA PCR kit (Life Technologies Italia, Italy) as previously described (Caputi et al., 2019, 2021). The qRT-PCR analysis was performed on a StepOne Real-Time PCR System (Life Technologies, Italy) using the SYBR Green PCR MasterMix (Life Technologies, Italy). Relative expression of different gene transcripts was calculated by the Delta-Delta Ct (ΔΔCt) method and converted to relative expression ratio (2^–ΔΔCt^) for statistical analysis (Livak and Schmittgen, 2001). All data were normalized to the housekeeping gene glyceraldehyde-3-phosphate dehydrogenase (*Gapdh*). The specificity of each PCR product was determined by melting curve analysis, constructed in the range of 60°C to 95°C. Primers used for PCR amplification were designed using Primer 3, and their sequences are reported as follows:

– *Gapdh* Forward 5′-AGACAGCCGCATCTTCTTGT-3′; Reverse 5′-CTTGCCGTGGGTAGAGTCAT-3′;
– *Nr3c1* Forward 5′-GAAAAGCCATCGTCAAAAGGG-3′ Reverse 5′-TGGAAGCAGTAGGTAAGGAGA-3′;
– *Cb1r* Forward 5′-GTCGATCCTAGATGGCCTTGC-3′; Reverse 5′-GTCATTCGAGCCCACGTAGAG-3′;
– *Faah* Forward 5′-GTTACAGAGTGGAGAGCTGTCC-3′; Reverse 5′-GTCTCACAGTCGGTCAGATAGG.

#### 2.5.3 Preparation of protein extracts and western blot analysis

Prefrontal cortex tissues were homogenized in a glass–glass potter using a cold buffer containing 0.32 M sucrose, 1 mM Hepes solution, 0.1 mM EGTA, 0.1 mM PMSF, pH = 7.4, in presence of a complete set of protease inhibitors and a phosphatase inhibitor cocktail, as previously described (Mottarlini et al., 2022). In brief, an aliquot of each homogenate was sonicated and then stored at −20°C, while the remaining homogenized tissues were centrifuged at 800 *g* for 10 min. The resulting pellet corresponding to the nuclear fraction, was resuspended in a buffer containing 20 mM Hepes, 0.1 mM DTT, 0.1 mM EGTA, with protease and phosphatase inhibitors. The supernatant was centrifuged at 12,000 *g* for 15 min to obtain the pellet corresponding to the crude synaptosomal fraction, and the resulting supernatant corresponds to a clarified fraction of cytosolic proteins. Total proteins have been measured in the nuclear, crude synaptosomal and cytosolic fractions by the Bio-Rad Protein Assay, using bovine serum albumin as the calibration standard (Bio-Rad Laboratories, Segrate, Milan, Italy). 10 micrograms of proteins for each sample were run on an SDS-10% polyacrylamide gel under reducing conditions and then electrophoretically transferred onto nitrocellulose membranes (Bio-Rad Laboratories). Blots were blocked 1 h at room temperature with 10% BSA or with I-Block solution (Life Technologies Italia) in TBS + 0.1% Tween-20 buffer, washed with TBS + 0.1% Tween-20 buffer and then incubated with the antibody anti-GR (1:500, RRID:AB_2283110, Thermo Scientific, USA); anti-CBD1 (1:1000, RRID:AB_10859098, ProteinTech, USA); anti-FAAH (1:1000, RRID:AB_2101994, Cell Signaling, USA). Results were standardized using β-actin (1:10.000, RRID:AB_476744, Sigma- Aldrich, Milan, Italy) as the control protein, which was detected by evaluating the band density at 43 kDa. Immunocomplexes were visualized by chemiluminescence using the Chemidoc MP Imaging System (Bio-Rad Laboratories) and analyzed using the Image Lab software (Bio-Rad Laboratories). Gels were run 2 times each and the results represent the average from 2 different western blots, averaged and normalized by using a specific correction factor (Caffino et al., 2020). In Supplementary Figure 1, results of GR protein levels measured individually in the nuclear and cytosolic fraction are presented. Full-size original cropped immunoblots related to the protein expression levels of GR, CB1R, FAAH are presented in Supplementary Figures 2–4 and representative immunoblots of the observed targets are shown in Figures 4–6.

### 2.6 Data analysis

Behavioral and biochemical data have been initially evaluated by Shapiro-Wilk tests to confirm the normality of the distribution and by Grubb’s test to identify outliers. Behavioral experiments were scored in a blinded conditions using the Observer 3.0 software (Noldus Information Technology, NL) and analyzed by Student *t*-test, Mann-Whitney test or two-way ANOVA. Molecular data were analyzed by two-way ANOVA and followed by Tukey’s multiple comparison test when appropriate. For the statistical analysis, the GraphPad Prism 9 software was used. Results are expressed as mean ± standard error of the mean (SEM). The level of significance was set at *p* < 0.05.

## 3 Results

### 3.1 Behavioral results

#### 3.1.1 Maternal behavior

Dams assigned to the SH group displayed a higher frequency of maternal behaviors compared to dams assigned to the CN group (*U* = 21.50; *p* = 0.0095) (Figure 1B). Conversely, mothers from the CN group showed a higher frequency of non-maternal behaviors in comparison to mothers of the SH group (*U* = 25.5; *p* = 0.0211) (Figure 1C), indicating that the two nesting conditions affected maternal care.

#### 3.1.2 Ultrasonic vocalizations

**FIGURE 2:**
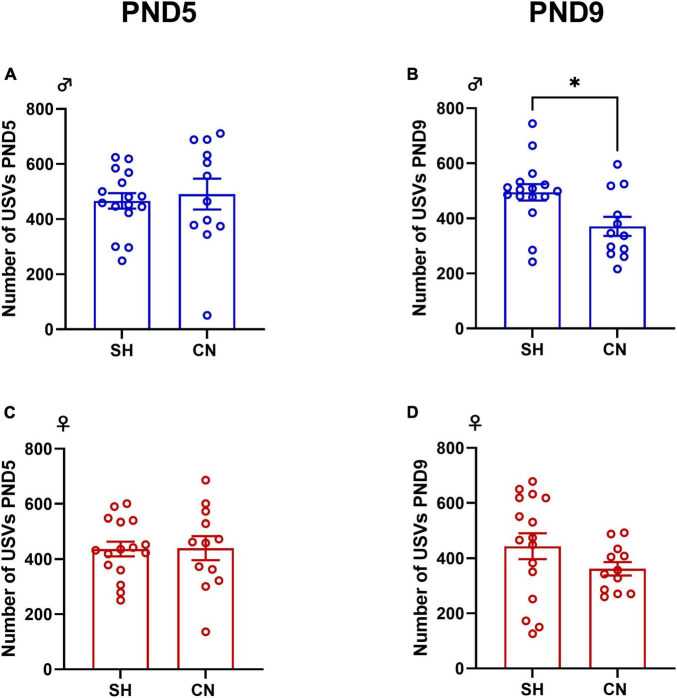
Effect of communal nesting on isolation-induced USVs in the infant rat offspring. At PND 5, no differences were found in the number of USVs emitted by the male **(A)** and female **(C)** offspring. At PND 9, CN male pups vocalized less compared to SH pups **(B)** while no differences were found in female rats **(D)** (Male: PND 5: SH = 16, CN = 12; PND 9: SH = 16, CN = 12; Female: PND 5: SH = 16, CN = 12; PND 9: SH = 16, CN = 12). Data represent mean ± S.E.M. *p < 0.05 vs. SH (Student’s *t*-test).

No differences between groups were found in the number of USVs emitted by male and female pups when separated from the dam at PND 5 (males: *t* = 0.41, df = 26, *p* = 0.6787; females: *t* = 0.057, df = 26, *p* = 0.9549) (Figures 2A, C). At PND 9, male pups from the CN group emitted less USVs when separated from the dam compared to male pups from the SH-group (*t* = 2.72, df = 26; *p* = 0.0114) (Figure 2B), whereas no significant differences were found in female pups (*t* = 1.41, df = 26; *p* = 0.1699) (Figure 2D).

#### 3.1.3 Elevated plus-maze test

**FIGURE 3:**
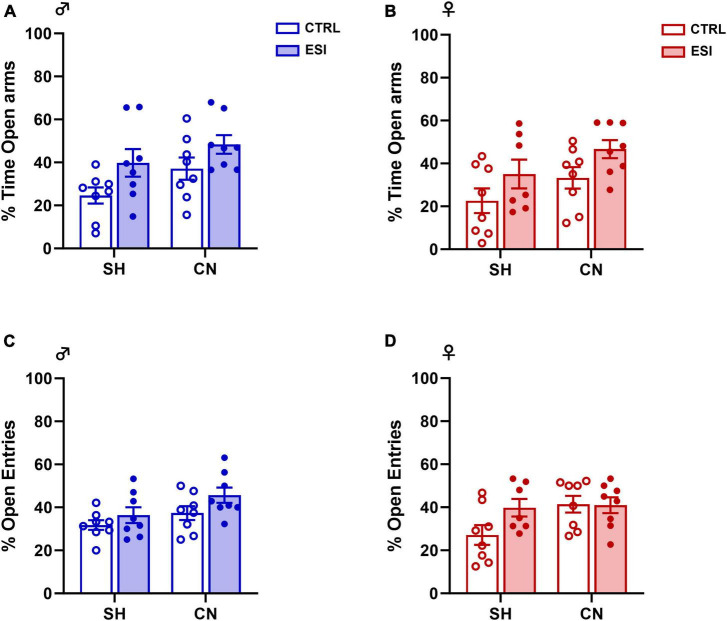
Effects of early social isolation (ESI) and communal nesting (CN) on the percentage (%) of time in the open arms **(A,B)** and on the percentage (%) of open arm entries **(C,D)** of male (left panels) and female (right panels) adolescent rats in the EPM test. No differences among groups were found in the percentage of time in the open arms **(A,B)** of the maze and in the percentage of entries into the open arms of the maze **(C,D)** (Males: SH-CTRL = 8, SH-ESI = 8, CN-CTRL = 8, CN-ESI: 8; Females: SH-CTRL = 8, SH-ESI = 7, CN-CTRL = 8, CN-ESI: 8). Data represent mean ± S.E.M. (Two-way ANOVA).

The two-way ANOVA analysis of the parameters measured in the elevated plus-maze test, performed in the adolescent male and female offspring, gave the following results:

– percentage of time spent in the open arms of the elevated plus-maze apparatus: males [*F*(ESI)_1,28_ = 6.93, *p* = 0.0136; *F*(CN)_1,28_ = 4.43, *p* = 0.0443; *F*(ESI x CN)_1,28_ = 0.15, *p* = 0.6996], (Figure 3A); females [*F*(ESI)_1,27_ = 5.70, *p* = 0.0243; *F*(CN)_1,27_ = 4.24, *p* = 0.0492; *F*(ESI x CN) _1,27_ = 0.007, *p* = 0.9325], (Figure 3B).
– percentage of open arm entries: males [*F*(ESI)_1,28_ = 4.03, *p* = 0.0544; *F*(CN)_1,28_ = 5.34, *p* = 0.0284; *F*(ESI x CN)_1,28_ = 0.34, *p* = 0.5624], (Figure 3C); females [*F*(ESI)_1,27_ = 2.22, *p* = 0.1476; *F*(CN)_1,27_ = 3.60, *p* = 0.0687; *F*(ESI x CN)_1,27_ = 2.59, *p* = 0.1190], (Figure 3D).

No differences in locomotion were observed, as no significant differences were detected in the total number of entries in both males [*F*(ESI)_1,28_ = 0.34, *p* = 0.559; *F*(CN)_1,28_ = 2.73, *p* = 0.1099; *F*(ESI x CN)_1,28_ = 0.57, *p* = 0.457] and females [*F*(ESI)_1,27_ = 0.18, *p* = 0.674; *F*(CN)_1,27_ = 2.16, *p* = 0.152; *F*(ESI x CN)_1,27_ = 0.17, *p* = 0.677]. Overall, these results indicate that the combination of nesting (SH or CN) and early social isolation (CTRL or ESI) conditions did not affect anxiety-like behavior in the elevated plus-maze test in the adolescent male and female rat offspring. Interestingly, similar results were found when the male and female offspring was tested in the elevated plus-maze at adulthood (See Supplementary material).

### 3.2 Biochemical results

#### 3.2.1 Plasma corticosterone levels

**FIGURE 4:**
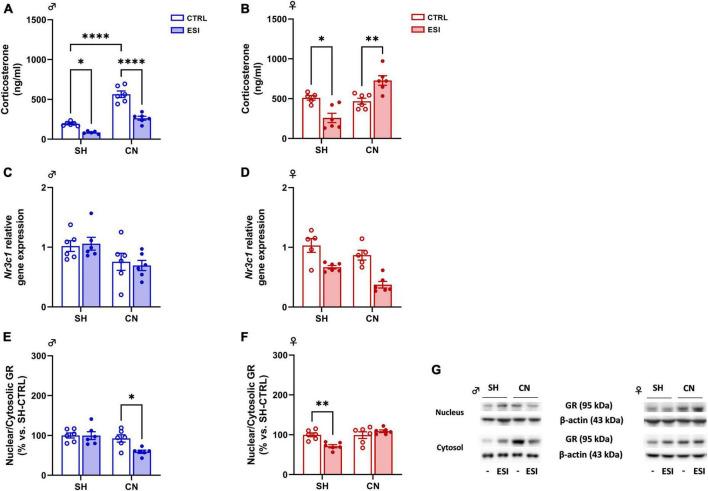
Effect of early social isolation (ESI) and different housing conditions on plasma corticosterone levels, *Nr3c1* gene expression, and GR protein expression in the PFC of adolescent male **(A,C,E)** and female **(B,D,F)** rats. Plasma corticosterone data **(A,B)** are expressed as ng/ml of mean ± SEM (*n* = 5–6/group; 1 outlier in SH-ESI males and 1 outlier in SH-CTRL females). Gene expression data **(C,D)** represent 2^–ΔΔCt^ values calculated by the ΔΔCt method. GR protein levels are shown as a ratio between nuclear and cytosolic fraction **(E,F)**. Protein data are expressed as percentages vs. SH-CTRL male or female rats. Histograms represent the mean ± SEM (*n* = 5–6/group; 1 outlier in SH-CTRL females and 1 outlier in CN-CTRL females). Data were analyzed by two-way ANOVA followed by Tukey’s multiple comparisons test (*p < 0.05, **p < 0.001, ****p < 0.0001). Representative immunoblots for GR protein levels (95 kDa) and β-Actin (43 kDa) are shown in panel **(G)**.

To investigate whether a combination of nesting (SH or CN) and early isolation (CTRL or ESI) conditions might affect the stress response of the HPA axis in adolescent offspring, we measured the circulating level of corticosterone in PND35 male and female offspring. In males, two-way ANOVA revealed a significant effect of CN [*F*_(1,19)_ = 112.7; *p* < 0.0001], of ESI [*F*_(1,19)_ = 62.21; *p* < 0.0001] and of CN x ESI interaction [*F*_(1,19)_ = 13.63; *p* = 0.0015] was observed (Figure 4A). *Post hoc* comparisons indicated that CN exposure in male rats increases corticosterone levels (+ 210 ng/ml vs SH-CTRL, *p* < 0.0001), while the ESI procedure reduced corticosterone levels in SH male rats (−55 ng/ml vs SH-CTRL, *p* = 0.0423), and in male rats previously exposed to CN (−141 ng/ml vs CN-CTRL, *p* = 0.0423). In female rats, two-way ANOVA showed a significant effect of CN [*F*_(1,19)_ = 18.30; *p* = 0.0004] and CN x ESI interaction [*F*_(1,19)_ = 26.27; *p* < 0.0001] whereas no effect of ESI [*F*_(1,19)_ = 0.0058; *p* = 0.9403] was observed (Figure 4B). Interestingly, *post hoc* comparisons showed a significant reduction of CORT in SH-ESI rats (−253 ng/ml vs SH-CTRL, *p* = 0.0120), while an increase of CORT was observed in ESI rats previously exposed to CN (+ 260 ng/ml vs CN-CTRL, *p* = 0.0065).

#### 3.2.2 Gene and protein expression in PFC

To assess the effects of different rearing conditions (SH or CN) and early isolation (CTRL or ESI) on GR, CB1R and FAAH expression we evaluated their mRNA and protein levels in the PFC of male and female offspring at PND35.

##### 3.2.2.1 Glucocorticoid receptor (GR)

*Gene expression:* In male adolescent rats, two-way ANOVA indicated a significant effect of CN [*F*_(1,20)_ = 8.293; *p* = 0.0093] on *Nr3c1* (gene encoding for GR) expression. However, no significant effect of ESI [*F*_(1,20)_ = 0.01018; *p* = 0.9206] and of CN x ESI interaction [*F*_(1,20)_ = 0.2147; *p* = 0.6481] was observed (Figure 4C). In females, the ANOVA revealed an overall effect of CN [*F*_(1,18)_ = 9.814; *p* = 0.0058] and of ESI [*F*_(1,18)_ = 34.88; *p* < 0.0001], but no significant effect of CN x ESI interaction was found [*F*_(1,18)_ = 0.8065; *p* = 0.3810] (Figure 4D).

*Protein expression:* To measure the level of GR translocation from the cytoplasm to the nucleus we performed a ratio of GR protein levels in the two subcellular fractions nucleus/cytosol, which is an index of GR nuclear internalization. In males, two-way ANOVA revealed significant effect of ESI [*F*_(1,20)_ = 4.790; *p* = 0.0407], CN [*F*_(1,20)_ = 9.918; *p* = 0.005] and CN x ESI interaction [*F*_(1,20)_ = 4.569; *p* = 0.0451] (Figure 4E). Interestingly, ESI reduced GR translocation only in CN rats (−33% vs CN-CTRL, *p* = 0.0291, −40% vs SH-ESI, *p* = 0.0065). In females, two-way ANOVA of GR nucleus/cytosol ratio revealed significant effect of CN housing [*F*_(1,20)_ = 11.28; *p* = 0.0031] and CN x ESI interaction [*F*_(1,20)_ = 12.54; *p* = 0.0021] whereas no effect of ESI [*F*_(1,20)_ = 2.889; *p* = 0.1047] was detected (Figure 4F). *Post hoc* comparisons revealed an ESI-induced reduction only in SH animals (−29% vs SH-CTRL, *p* = 0.007; −38% vs CN-ESI, *p* = 0.0005).

##### 3.2.2.2 Cannabinoid receptor type-1 (CB1R)

**FIGURE 5:**
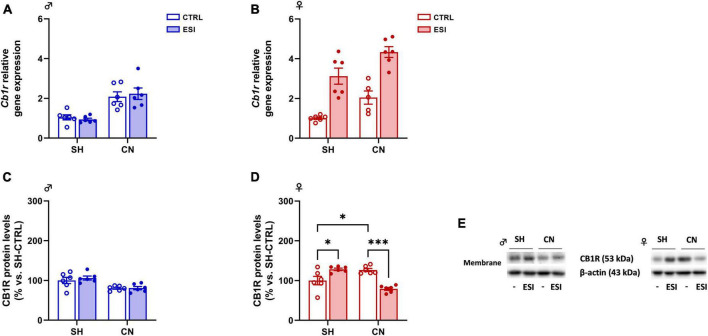
Effect of early social isolation (ESI) and different housing conditions on CB1R gene and protein expression in the PFC of adolescent male **(A,C)** and female **(B,D)** rats. Gene expression data **(A,B)** represent 2^–ΔΔCt^ values calculated by the ΔΔCt method and are expressed as mean ± SEM (*n* = 5–6/group; 1 outlier in CN-CTRL females). Protein levels **(C,D)** are shown in the cortical membrane fraction. Data are expressed as percentages of SH-CTRL male and female rats, respectively. Histograms represent the mean ± SEM (*n* = 6/group). Data were analyzed by two-way ANOVA followed by Tukey’s multiple comparisons test (*p < 0.05, ***p < 0.001). Representative immunoblots for CB1R protein levels (53 kDa) and β-Actin (43 kDa) are shown in panel **(E)**.

*Gene expression:* Two-way ANOVA of *Cb1r* gene expression indicated a significant effect of CN [*F*_(1,20)_ = 33.08; *p* < 0.0001] in adolescent male rats. However, no significant effect of ESI [*F*_(1,20)_ = 0.01053; *p* = 0.9193] and of CN x ESI interaction [*F*_(1,20)_ = 40.31; *p* = 0.5327] was observed (Figure 5A). In adolescent female rats, overall ANOVA revealed a main effect of CN [*F*_(1,19)_ = 14.47; *p* = 0.0012] and of ESI [*F*_(1,19)_ = 55.31; *p* < 0.0001], however, no significant effect of CN x ESI interaction [*F*_(1,19)_ = 0.08820; *p* = 0.7697] was observed on *Cb1r* gene expression (Figure 5B).

*Protein levels:* In male rats, two-way ANOVA of cortical CB1R protein expression revealed a significant effect of CN [*F*_(1,20)_ = 18.58; *p* = 0.0003] whereas no effect of ESI [*F*_(1,20)_ = 0.3987; *p* = 0.5349] and of CN x ESI interaction [*F*_(1,20)_ = 0.3122; *p* = 0.5825] was observed (Figure 5C). In female rats, two-way ANOVA revealed only a significant effect of CN x ESI interaction [*F*_(1,20)_ = 37.61; *p* < 0.0001; ESI: *F*_(1,20)_ = 2.349; *p* = 0.5349; CN: *F*_(1,20)_ = 3.343; *p* = 0.5825; Figure 5D]. Further intergroup sub-testing showed that CN *per se* increased CB1R expression (+ 27% vs SH-CTRL, *p* = 0.03). Interestingly, ESI altered CB1R expression differently depending on the housing condition. In fact, while ESI increased CB1R expression in SH rats (+ 28% vs SH-CTRL, *p* = 0.0192), ESI reduced its expression in CN animals (−47% vs CN-CTRL, *p* = 0.0001, −49% vs SH-ESI, *p* < 0.0001).

##### 3.2.2.3 Fatty acid amide hydrolase (FAAH)

**FIGURE 6:**
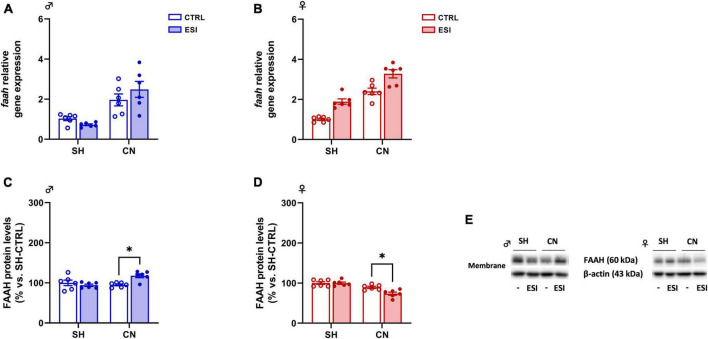
Effect of early social isolation (ESI) and different housing conditions on FAAH gene expression in the PFC of adolescent male **(A,C)** and female **(B,D)** rats. Gene expression data **(A,B)** represent 2^–ΔΔCt^ values calculated by the ΔΔCt method and are expressed as mean ± SEM (*n* = 5–6/group). Protein levels **(C,D)** are shown in the cortical membrane fraction. Data are expressed as percentages of SH-CTRL male and female rats, respectively. Histograms represent the mean ± SEM (*n* = 6/group). Data were analyzed by two-way ANOVA followed by Tukey’s multiple comparisons test (*p < 0.05). Representative immunoblots for FAAH protein levels (63 kDa) and β-Actin (43 kDa) are shown in panel **(E)**.

*Gene expression:* In adolescent male rats, two-way ANOVA indicated a significant effect of CN [*F*_(1,20)_ = 28.21; *p* < 0.0001] on *Faah* gene expression. However, no significant effect of ESI [*F*_(1,20)_ = 0.1832; *p* = 0.6732] and of CN x ESI interaction [*F*_(1,20)_ = 2.683; *p* = 0.1171] was observed (Figure 6A). The overall ANOVA analysis displayed a main effect of CN [*F*_(1,20)_ = 87.74; *p* < 0.0001] and of ESI [*F*_(1,20)_ = 35.27; *p* < 0.0001] on FAAH gene expression in female rats. While, no significant effect of CN x ESI interaction [*F*_(1,20)_ = 0.001856; *p* = 0.9661] was revealed (Figure 6B).

*Protein expression:* In male rats, two-way ANOVA of cortical FAAH protein expression revealed a significant effect of CN x ESI interaction [*F*_(1,20)_ = 9.390; *p* = 0.0061] whereas no effect of ESI [*F*_(1,20)_ = 3.448; *p* = 0.0781] and of CN [*F*_(1,20)_ = 3.855; *p* = 0.0637] was observed (Figure 6C). *Post hoc* comparisons indicate that ESI increased FAAH protein levels only in CN rats (+ 24% vs SH-ESI, *p* = 0.0098; + 23% vs CN-CTRL, *p* = 0.0116).

In female rats, two-way ANOVA revealed a significant effect of ESI [*F*_(1,20)_ = 5.944; *p* = 0.0242], CN [*F*_(1,20)_ = 32.38; *p* < 0.0001] and CN x ESI interaction [*F*_(1,20)_ = 5.616; *p* = 0.028; Figure 6D]. Opposite to male rats, in female PFC ESI reduced FAAH protein levels only in CN animals (−26% vs SH-ESI, *p* < 0.0001; −15% vs CN-CTRL, *p* = 0.0139).

## 4 Discussion

The present study investigated the impact of early social environment on the emotional reactivity of adolescent male and female rats, focusing on the cortical eCB and glucocorticoid systems. To this aim, we applied a protocol of environmental manipulation based on early postnatal socially enriched or impoverished conditions. The ability of the environmental manipulations used in our study (ESI and CN) to induce physiological and emotional changes in rodents have been previously demonstrated, although most of the available studies assessed their impact at adult age. For instance, it has been shown that mice exposed to the same ESI protocol used in our study (i.e., single housing in a cage with clean bedding for 30 min/day from PND 14 to PND 21) showed depressive-like behaviors at adulthood (Lo Iacono et al., 2015) associated with epigenetic changes in different brain regions (Catale et al., 2020). Furthermore, we recently showed that that the same ESI protocol used in our study did not affect locomotor activity and the time spent in the central part of the open field arena in the adolescent rat offspring, although ESI altered sensorimotor gating and burying behavior (Bratzu et al., 2023), thus supporting the idea that this ESI protocol induces subtle behavioral changes in the rat offspring. Similarly, it has been shown that CN in mice affected depressive- and anxiety-like responses later in life (Branchi and Alleva, 2006; Branchi, 2009; Cirulli et al., 2010), although less information is available on the impact of CN in adolescent animals. For instance, adult male mice reared in the CN condition displayed a higher propensity for social interaction (Branchi et al., 2006a) and they showed anxiety-like behaviors in the elevated plus-maze and open field tests (Branchi et al., 2006b). Interestingly, however, adult female mice reared in the CN condition did not differ in the emotional responses measured in the zero-maze apparatus (Gracceva et al., 2009). Furthermore, other studies (Sayler and Salmon, 1971) failed to detect changes in emotional reactivity in adult CN mice. Based on these controversial findings and on the paucity of data about the impact of early environmental manipulations in developing animals of both sexes, we assessed the impact of ESI and CN in the rat male and female offspring during adolescence, a critical time window for brain maturation.

Our results showed that the two forms of social manipulation (i.e., communal nesting, CN and early social isolation, ESI) induced different behavioral and molecular outcomes in female and male rats. Molecular results underline that the environmental manipulations resulted in distinct alterations in mRNA and protein expression of the main components of the glucocorticoid and eCB systems. Interestingly, elevated plasma CORT levels together with reduced CB1R and FAAH protein expression have been detected in CN-ESI females. However, an opposite picture has been found in the corresponding male group, thus suggesting that the previous exposure to CN differently affected ESI-induced molecular alterations in the two sexes. Actually, significant sex-dependent effects in response to an early life stress have been previously reported in both the glucocorticoid (Pallarés et al., 2021) and the eCB (Dow-Edwards, 2020) systems.

It has been suggested that CN provides a highly stimulating social environment to the developing pups because they are exposed to diverse styles of maternal care and to a higher number of peer-peer interactions (Branchi and Cirulli, 2014). This social stimulation is supposed to have a major impact on brain function and behavioral development (Branchi, 2009). In a synchronous CN (all females give birth on the same day), the total amount of maternal behavior displayed by the mothers in the CN condition has been reported to be greater compared to standard laboratory rearing conditions (Sayler and Salmon, 1971; Branchi et al., 2006a,b). Here, we found that rat dams assigned to the CN procedure showed reduced frequency of maternal behaviors compared to dams in the SH condition. In line with our findings, some studies reported reduced maternal behavior after prenatal exposure to environmental enrichment (Cancedda et al., 2004; Welberg et al., 2006; Rosenfeld and Weller, 2012), or CN housing (Gracceva et al., 2009). Differences between mice and rats in their response to environmental manipulations during pregnancy and lactation, as well as differences in the CN protocol applied, may account for these discrepant findings.

In the present study, the reduced maternal behavior displayed by CN dams could impact on the emotional reactivity of the pups and their relationship with the dam.

We tested pups’ emotionality by measuring their ultrasonic vocalization (USV) emission following a brief period of isolation from the mother and siblings. These USVs play an important role in mother–offspring interactions and are indeed an indicator of emotional reactivity in the pups representing a potent tool used to detect subtle effects of adverse events during development. We found that nesting condition did not affect pup USV emission at PND5. Conversely, at PND9 male, but not female, pups reared in the CN emitted a lower number of USVs when separated from the mother and littermates. This result is in line with previous studies showing that mouse pups born from dams housed together from the day of parturition (a condition referred to as “communal rearing”) (Sayler and Salmon, 1971; Curley et al., 2009) display reduced basal USV production (Cirulli et al., 2010), although this study did not find differences between the USV rate of male and female pups. The reduced number of USVs emitted by male pups born in the CN condition may be related to a reduced anxious-like phenotype compared to pups born in the SH condition.

To mimic early social deprivation, each pup either reared in CN or SH conditions was subjected to an early social isolation (ESI) protocol from PND 14 to PND 21. The emotional reactivity of the animals was assessed at adolescence by the elevated plus-maze (EPM) test. We found no changes between both male and female rats subjected to CN protocol and then exposed to the ESI procedure. These unexpected results could indicate that, despite these pups were exposed to reduced maternal care between PND2 and PND13, other factors such as a high number of peer-peer interactions or increased maternal care after the ESI protocol, may impact the development of their emotional behavior (Branchi and Alleva, 2006). Moreover, the long-term impact of CN on the emotional reactivity of the offspring seems to be dependent on which facet of emotionality is assessed, e.g., social anxiety versus exposure to a physical challenge (Branchi et al., 2006a,b), but it is also dependent on the CN protocol used (Andersen, 2003; Sparling et al., 2020). For instance, it has been reported that, at adulthood, the CN offspring show greater emotionality in the open-field and in the elevated plus-maze (but not in a social interaction test) when the mothers are placed together 5 days before parturition (Branchi et al., 2006a,b; Branchi, 2009), while opposite effects are observed when the mothers are put together on the day of parturition (Sayler and Salmon, 1971; Curley et al., 2009). Altogether, the results of the isolation-induced USV and EPM tests suggest that early social manipulations may induce sex-dependent changes in emotional behavior, indicated by a reduced USV rate, that can already be detected during the first days of life but then normalize at a later age. Alternatively, it is still possible that subtle changes in anxiety-like behaviors could be detected in adolescent and adult CN-exposed animals if behavioral tasks other than the elevated plus-maze test were performed.

From a molecular point of view, in the last decade, several lines of evidence indicate that the eCB system is involved in the central regulation of the stress response to life challenges, such as early life events; indeed, eCBs are involved in glucocorticoid-feedback inhibition of the HPA axis in the PFC, which represents a neuroanatomical site integral for delayed feedback inhibition (mediated by GR translocation into the nucleus) of the HPA axis. In this scenario, corticosterone represents the major stress hormone and it is known to play a regulatory role in stress induced HPA axis activity in rodents. Elevated corticosterone levels activate the stress-response network and affect various neural circuits involved in stress-coping (Avishai-Eliner et al., 2001; Kinlein et al., 2019).

In our model, adolescent female rats exposed to the combination of CN and ESI, showed increased corticosterone plasma levels, unaltered genomic GR response (as shown by the unaffected nucleus/cytosol ratio) and reduced CB1R and FAAH protein levels. Even though ESI exposure in CN reared female rats increases CORT levels, the corticosterone-induced negative feedback on the HPA axis is not effective because GR translocation into the nucleus is not altered, suggesting that the nuclear GR is unresponsive to the increased levels of the glucocorticoid. Moreover, in these rats, the glucocorticoid actions mediated by the increased CORT levels might have indeed induced a stimulation of the eCB system not through the *de novo* synthesis of eCBs but, rather, via reducing the endocannabinoid degrading enzyme FAAH which, in turn, increases eCBs levels in the PFC. However, since CB1R protein levels were significantly reduced, the hypothesized increase of endocannabinoids concentrations is potentially unable to foster the glucocorticoid-feedback inhibition of the HPA axis. Accordingly, human studies suggested that permanently elevated anandamide levels together with early life stress may cause a lifelong damage on stress response probably through CB1R reduction during neurodevelopment (Lazary et al., 2016).

We speculate that the inability to respond to corticosterone observed in CN female rats exposed to ESI might be due to the increased levels of endocannabinoids which, via a non-CB1R dependent mechanism, may dysregulate the HPA axis ability to respond to negative stimuli.

Despite several evidence suggested that FAAH inhibition may be a valid pharmacological strategy to reduce HPA axis activation and elicit anxiolytic/antidepressant effects (Haller et al., 2009; Carnevali et al., 2020), it has also been shown that the efficacy of this treatment strongly depends on the developmental time window of treatment (Alteba et al., 2021). Indeed, FAAH inhibition may have deleterious or ameliorating effects on behavior depending on its mid- or late-adolescence occurrence.

The dysregulation of the eCB signaling following the combination of CN and ESI in female rats is further underlined by the uncoupling between mRNA and protein levels for both CB1R and FAAH. According with previous evidence (Demaili et al., 2023), the ESI-induced long lasting up-regulation of *Cb1r* and *Faah* mRNA levels may alter excitation/inhibition balance in the PFC of adolescent females thus affecting their ability to cope with stress later in life.

Conversely to what observed in females, male CN rats exposed to ESI show reduced corticosterone plasma levels and GR nuclear translocation, suggesting that CN rearing condition positively influence the development of important behavioral competences useful to cope with social challenges, thus blunting CN-induced HPA axis activity. Moreover, in light of our glucocorticoid results and despite no changes were observed in CB1R protein levels, it is possible that the CN-induced increase of *Cb1r* gene expression could represent an adaptive mechanism aimed to reduce HPA axis activity (Lazary et al., 2016), as we indeed observed. In addition, the FAAH enzyme increases in these animals, possibly leading to a decrease in eCBs levels, an effect that could influence HPA axis activation reducing endocannabinoid inhibitory tone in this brain region (Hill and McEwen, 2010; Hill and Tasker, 2012). Such a reduction corroborates our hypothesis of reduced HPA activity, since it might have potentiated the negative feedback loop of HPA axis, as shown by the reduced levels of CORT. Indeed, in accordance with our findings, a lower vulnerability to acute or prolonged social stress consequences has been reported in CN- rather than in SH-reared mice (Branchi et al., 2010).

It is difficult to conclude whether each biochemical change here reported could have adaptive or maladaptive outcomes thus leading to vulnerability or resilience to stressors. However, also in the light of other investigations (see Viveros et al., 2012 for review), these results highlight that different early life experiences can produce a wide panel of neurochemical alterations, and that these alterations can be influenced by sex.

Even though this work provides novel findings about the effects of social manipulations early in life on the homeostasis of glucocorticoids and eCB system that may differentially affect male and females coping abilities, we are aware that it holds strengths as well as limitations. Indeed, it represents one of the first evidence showing the influence of CN on adverse events occurring later in life, through a mechanism that involves the coordinated action of glucocorticoids and eCBs. In addition, we observed differences between males and females exposed to CN in the modulation of the response to the early life stress, providing further evidence that maternal care influences the coping to emotional events in males and females, differently. However, a potential limitation may be represented by the fact that the core of our analyses was done during adolescence and, therefore, we cannot extend our considerations to adulthood. Furthermore, only the emotional domain of the offspring was assessed, leaving open doors to the possibility that these sex-dependent changes in the response to early social manipulations may affect other behavioral domains (e.g., motor, cognition, social behavior). In addition, the evaluation of sex differences in the HPA axis response by acute restraint stress and of CORT levels in rat hair could have added relevant information. Therefore, further investigations will be useful to elucidate these aspects.

In conclusion, we found that CN shapes the response to ESI in the rat PFC differently in males and females and that glucocorticoids and eCBs influence each other in the modulation of such a response.

## Data availability statement

The original contributions presented in the study are included in the article/Supplementary material, further inquiries can be directed to the corresponding author.

## Ethics statement

The animal study was approved by the Italian Ministry of Health Ethics Committee at Roma 3 University. The study was conducted in accordance with the local legislation and institutional requirements.

## Author contributions

LR: Data curation, Investigation, Methodology, Writing—original draft, Writing—review and editing. LML: Investigation, Methodology, Writing—original draft, Writing—review and editing. CM: Investigation, Methodology. FM: Data curation, Investigation, Methodology, Writing—original draft, Writing—review and editing. SS: Writing—review and editing, Investigation, Methodology. VB: Writing—review and editing, Investigation, Methodology. FA: Investigation, Methodology. RC: Conceptualization, Supervision, Funding acquisition, Writing—original draft, Writing—review and editing. LF: Conceptualization, Supervision, Funding acquisition, Writing—original draft, Writing—review and editing. LC: Data curation, Methodology, Investigation, Writing—original draft, Writing—review and editing. FF: Writing—original draft, Supervision, Writing—review and editing, Conceptualization, Funding acquisition. PR: Conceptualization, Funding acquisition, Supervision, Writing—original draft, Writing—review and editing. VT: Conceptualization, Supervision, Funding acquisition, Writing—original draft, Writing—review and editing. SC: Investigation, Supervision, Writing—original draft, Writing—review and editing.
