# Supplementary material for: Outcomes of early social experiences on glucocorticoid and endocannabinoid systems in the prefrontal cortex of male and female adolescent rats
Source: Front Cell Neurosci. 2023 Dec 20;17:1270195. doi: 10.3389/fncel.2023.1270195 (PMC10762649; doi:10.3389/fncel.2023.1270195)
Supplement: Supplementary file 1 [file Data_Sheet_1.pdf]

Supplementary Material

1.1 Elevated plus maze test in male and female adult (PND 75) SH and CN rats previously exposed to CTRL or ESI conditions.

The two-way ANOVA analysis of the parameters measured in the elevated plus-maze test performed in the adult male and female offspring gave the following results:

- percentage of time spent in the open arms of the elevated plus-maze apparatus: males ($F(ESI)_{1,26}$ = 1.053, p = n.s.; $F(CN)_{1,26}$ = 0.92, p = n.s.; $F(ESI \times CN)_{1,26}$ = 2.25, p = n.s.; females ($F(ESI)_{1,28}$ = 8.58, $p < 0.01$; $F(CN)_{1,28}$ = 0.14, p = n.s.; $F(ESI \times CN)_{1,28}$ = 0.07, p = n.s).

- percentage of open arm entries: males ($F(ESI)_{1,26}$ = 0.78, p = n.s.; $F(CN)_{1,26}$ = 0.74, p = n.s.; $F(ESI \times CN)_{1,26}$ = 2.86, p = n.s.; females: $F(ESI)_{1,28}$ = 4.57, $p < 0.05$; $F(CN)_{1,28}$ = 0.81, p = n.s.; $F(ESI \times CN)_{1,28}$ = 0.012, p = n.s.).

Overall, these results indicate that the combination of nesting (SH or CN) and early social isolation (CTRL or ESI) conditions did not affect anxiety-like behavior in the elevated plus-maze test neither in the adolescent nor in adult offspring. This finding is in line with the evidence that adolescent male and female rats exposed to the same ESI protocol used in the present study did not differ from control animals in the time spent in the central part of the open field arena (Bratzu et al., 2023), a parameter that is also often used to evaluate potential changes in anxiety-like behaviors in rodents. It is still possible, however, that subtle changes in anxiety-like behaviors could be detected if other behavioral tasks were performed, as also suggested by the recently reported finding of a significant effect of both ESI and housing condition observed in adolescence but not adulthood in rats reared under the same experimental conditions and tested in the marble burying test, i.e., a test widely used to assess anxiety-like state/neophobia in rodents (Bratzu et al., 2023).

1.2 Protein expression levels of glucocorticoid receptor measured in the cytosolic and nuclear fraction of SH and CN rats previously exposed to CTRL or ESI conditions.

In male rats, two-way ANOVA of GR protein expression in the cytosolic fraction revealed a significant effect of CN ($F_{(1, 20)}$ = 9.049; p = 0.0069) and CN x ESI interaction ($F_{(1, 20)}$ = 6.322; p = 0.0206) whereas no effect of ESI ($F_{(1, 20)}$ = 1.515; p = 0.2327) was observed (**Figure S1A**). *Post-hoc* comparisons indicated that only CN per se increased GR protein levels in CTRL rats (+51% vs SH-CTRL, p = 0.0045). In the nuclear fraction of male rats, two-way ANOVA showed a significant effect of CN x ESI interaction ($F_{(1, 20)}$ = 40.11; $p < 0.0001$) whereas no effect of ESI ($F_{(1, 20)}$ = 3.078; p = 0.0947) and CN ($F_{(1, 20)}$ = 0.7833; p = 0.3867) was observed (**Figure S1C**). CN per se increased GR nuclear expression in CTRL rats (+36% vs SH-CTRL, p = 0.0051). Interestingly, ESI differently altered GR expression depending on the housing condition. In fact, while ESI increased GR expression in SH rats (+30% vs SH-ESI, p = 0.0198), ESI reduced its expression in CN animals (-54% vs CN-CTRL, $p < 0.0001$ and -48% vs SH-ESI, p = 0.0003).

In female rats, two-way ANOVA of GR protein levels in the cytosolic fraction revealed a significant effect of ESI ($F_{(1, 20)}$ = 7.789; p = 0.0113), CN ($F_{(1, 20)}$ = 4.386; p = 0.0492) and CN x ESI interaction ($F_{(1, 20)}$ = 7.684; p = 0.0118; **Figure S1B**). Looking at the single contrasts, similar to male rats, CN increased cytosolic GR levels in females (+40% vs SH-CTRL, p = 0.0127). In addition, ESI increased GR protein levels in the cytosol only in SH animals (+46% vs SH-CTRL, p = 0.0042). Two-way ANOVA of GR protein expression in the nuclear fraction revealed a significant effect of CN ($F_{(1, 20)}$ = 21.98; p = 0.0001) whereas no effect of ESI ($F_{(1, 20)}$ = 1.371; p = 0.2554) and CN x ESI interaction ($F_{(1, 20)}$ = 0.4437; p = 0.5130) was observed (**Figure S1D**).

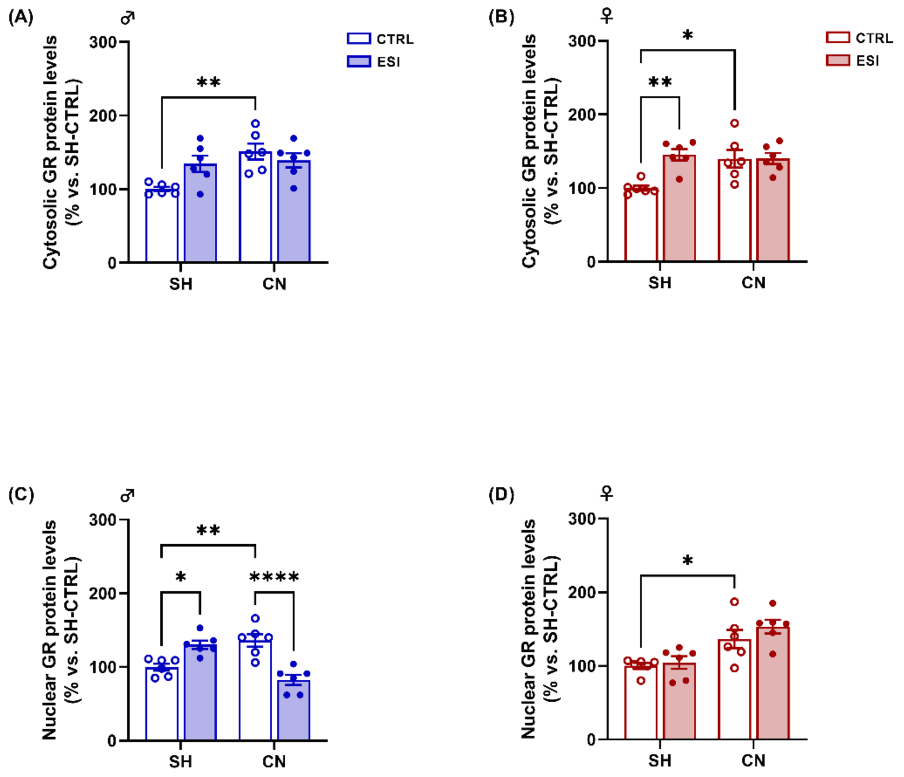

Supplementary Figure 1 (S1). Effect of early social isolation (ESI) and different housing conditions on GR protein expression in the PFC of adolescent male (A, C) and female (B, D) rats. GR protein levels are shown in the cytosolic fraction (panels A, B) and in the nuclear fraction (panels C, D). Protein data are expressed as percentages vs. SH-CTRL male or female rats. Below each graph, representative immunoblots are shown for GR protein levels (95 kDa) and β-Actin (43 kDa). Histograms represent the mean ± SEM (n = 5-6/group). Data were analyzed by two-way ANOVA followed by Tukey's multiple comparisons test (*p<0.05, **p<0.01, ****p<0.0001).

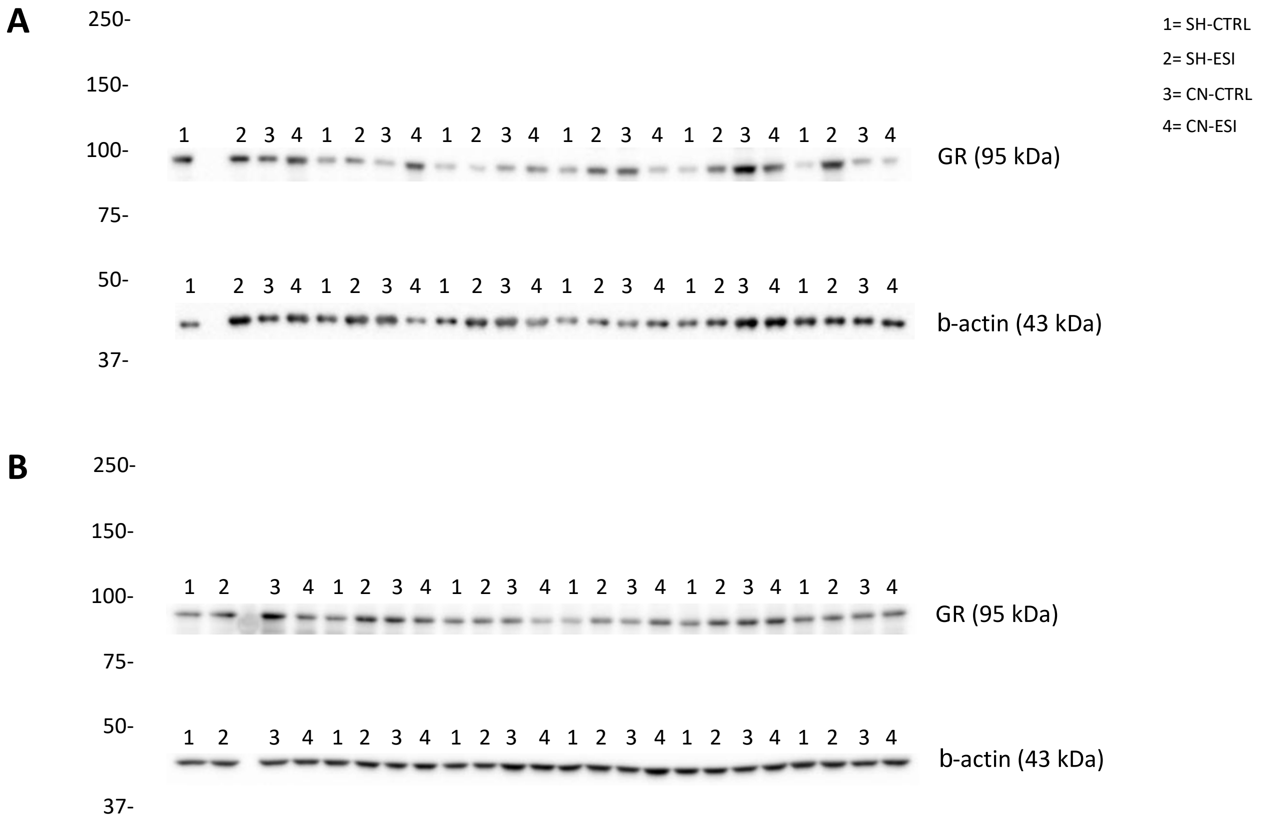

Supplementary Figure 2 (S2). Cropped immunoblot related to the expression levels of GR and β-actin measured in the cytosolic fraction of medium prefrontal cortex of SH-CTRL, SH-ESI, CN-CTRL and CN-ESI male (A) and female (B) rats, presented in in the manuscript (**Figure 4**).

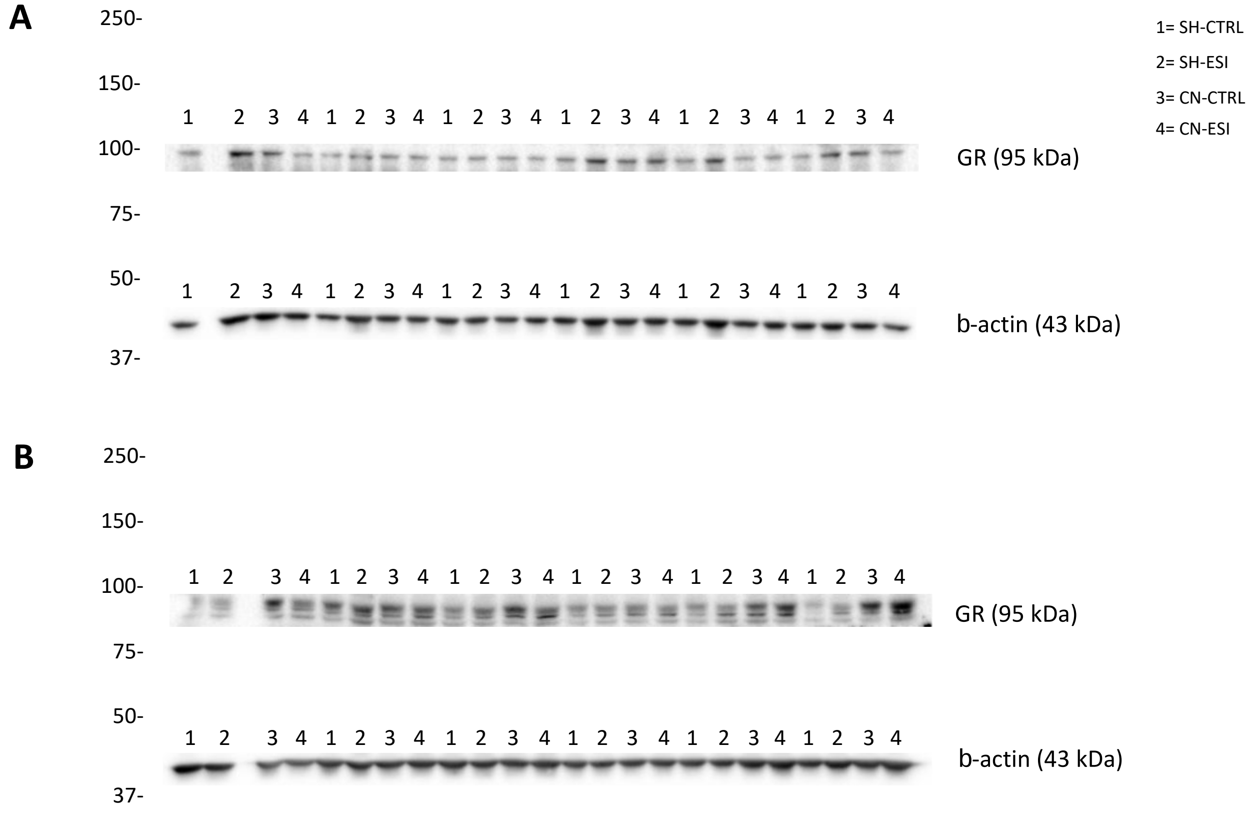

Supplementary Figure 3 (S3). Cropped immunoblot related to the expression levels of GR and β-actin measured in the nuclear fraction of medium prefrontal cortex of SH-CTRL, SH-ESI, CN-CTRL and CN-ESI male (A) and female (B) rats, as presented in the manuscript (**Figure 4**).

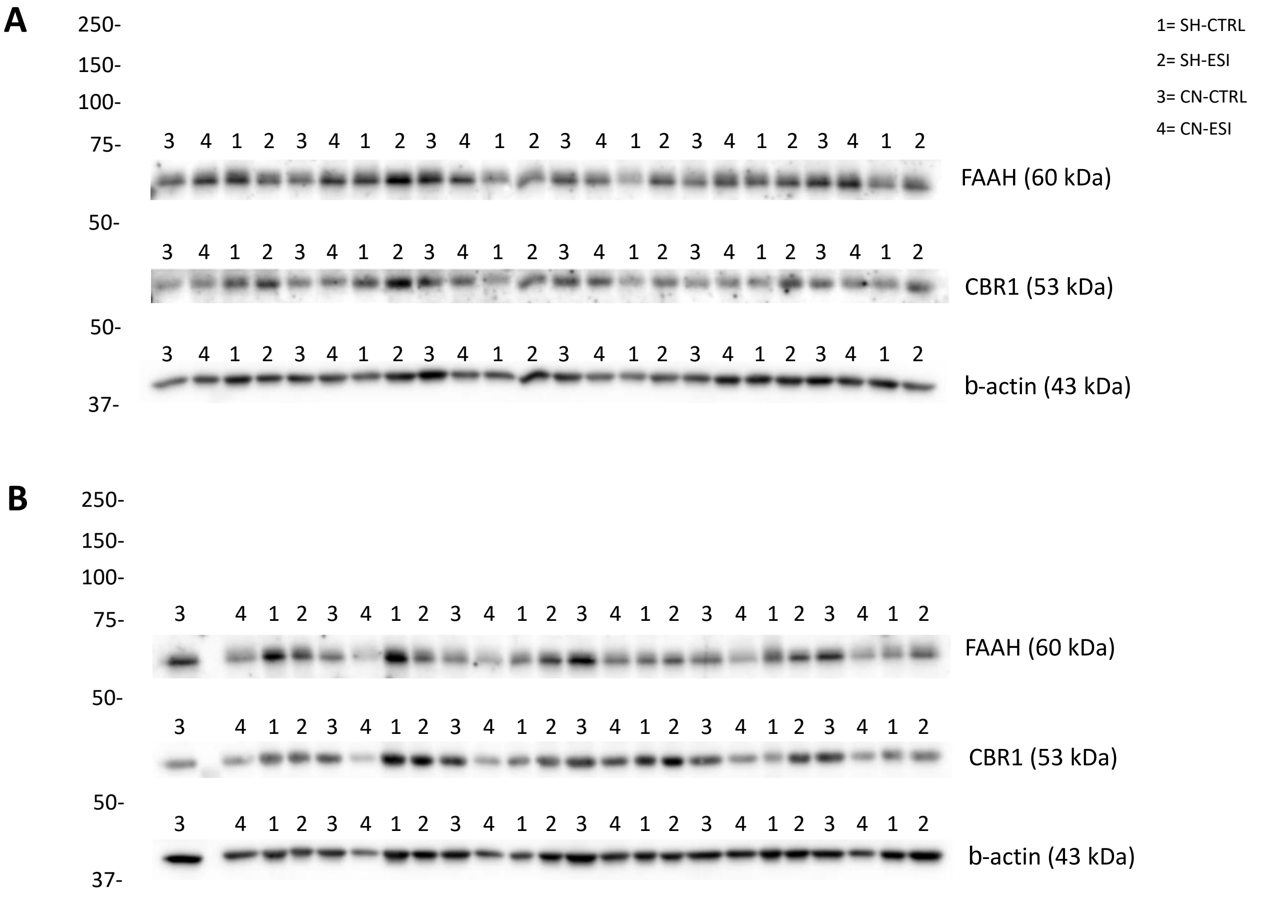

Supplementary Figure 4 (S4). Cropped immunoblot related to the expression levels of CBR1, FAAH and β-actin measured in the crude membrane fraction of medium prefrontal cortex of SH-CTRL, SH-ESI, CN-CTRL and CN-ESI male (A) and female (B) rats, presented in manuscript as **Figures 5** and **Figure 6**.

Supplementary Table 1. Elevated plus maze results (F values, degrees of freedom and p values) of two-way ANOVA analyses in male and female adolescent rats (PND 35) SH or CN rats previously exposed to CTRL or ESI conditions.

		Males			Females	
	**Two-way ANOVA**	**F value**	**p value**		**F value**	**p value**
**% of time spent in the open arms**	Communal Nesting (CN)	F (1,28) = 4.43	p = 0.0443		F (1,27) = 4.24	p = 0.0492
	Early Social Isolation (ESI)	F (1,28) = 6.93	p = 0.0136		F (1,27) = 5.70	p = 0.0243
	Interaction (CN X ESI)	F (1,28) = 0.15	p = 0.6996		F (1,27) = 0.007	p = 0.9325
**% of open arm entries**	Communal Nesting (CN)	F (1,28) = 5.34	p = 0.0284		F (1,27) = 3.60	p = 0.0687
	Early Social Isolation (ESI)	F (1,28) = 4.03	p = 0.0544		F (1,27) = 2.22	p = 0.1476
	Interaction (CN X ESI)	F (1,28) = 0.34	p = 0.5624		F (1,27) = 2.59	p = 0.1190
**number of total arm entries**	Communal Nesting (CN)	F (1,28) = 2.73	p = 0.1099		F (1,27) = 2,16	p = 0.152
	Early Social Isolation (ESI)	F (1,28) = 0.34	p = 0.559		F (1,27) = 0.18	p = 0.674
	Interaction (CN X ESI)	F (1,28) = 0.57	p = 0.457		F (1,27) = 0.17	p = 0.677

Supplementary Table 2. Biochemical results (F values, degrees of freedom and p values) of two-way ANOVA analyses in male and female adolescent rats (PND 35) SH or CN rats previously exposed to CTRL or ESI conditions.

		Males			Females	
	Two-way ANOVA	F value	p value		F value	p value
Plasma CORT levels	Communal Nesting (CN)	F (1,19) = 112.7	p < 0.0001		F (1,19) = 18.30	p = 0.0004
	Early Social Isolation (ESI)	F (1,19) = 62.21	p < 0.0001		F (1,19) = 0.0058	p = 0.9403
	Interaction (CN X ESI)	F (1,19) = 13,63	p = 0.0015		F (1,19) = 26.27	p < 0.0001
Nr3c1	Communal Nesting (CN)	F (1,20) = 8.293	p = 0.0093		F (1,18) = 9.814	p = 0.0058
	Early Social Isolation (ESI)	F (1,20) = 0.01018	p = 0.9206		F (1,18) = 34.88	p < 0.0001
	Interaction (CN X ESI)	F (1,20) = 0.2147	p = 0.6481		F (1,18) = 0.8065	p = 0.3810
**GR (nuclear / cytosolic fraction)**	Communal Nesting (CN)	F (1,20) = 9.918	p = 0.005		F (1,20) = 11.28	p = 0.0031
	Early Social Isolation (ESI)	F (1,20) = 4.790	p = 0.0407		F (1,20) = 2.889	p = 0.1047
	Interaction (CN X ESI)	F (1,20) = 4.569	p = 0.0451		F (1,20) = 12.54	p = 0.0021
Cb1r	Communal Nesting (CN)	F (1,20) = 33.08	p < 0.0001		F (1,19) = 14.47	p = 0.0012
	Early Social Isolation (ESI)	F (1,20) = 0.01053	p = 0.9193		F (1,19) = 55.31	p < 0.0001
	Interaction (CN X ESI)	F (1,20) = 40.31	p = 0.5327		F (1,19) = 0.08820	p = 0.7697
**CB1R**	Communal Nesting (CN)	F (1,20) = 18.58	p = 0.0003		F (1,20) = 3.343	p = 0.5825
	Early Social Isolation (ESI)	F (1,20) = 0.3987	p = 0.5349		F (1,20) = 2.349	p = 0.5349
	Interaction (CN X ESI)	F (1,20) = 0.3122	p = 0.5825		F (1,20) = 37.61	p < 0.0001
Faah	Communal Nesting (CN)	F (1,20) = 28.21	p < 0.0001		F (1,20) = 87.74	p < 0.0001
	Early Social Isolation (ESI)	F (1,20) = 0.1832	p = 0.6732		F (1,20) = 35.27	p < 0.0001
	Interaction (CN X ESI)	F (1,20) = 2.683	p = 0.1171		F (1,20) = 0.001856	p = 0.9661
**FAAH**	Communal Nesting (CN)	F (1,20) = 3.855	p = 0.0637		F (1,20) = 32.38	p < 0.0001
	Early Social Isolation (ESI)	F (1,20) = 3.448	p = 0.0781		F (1,20) = 5.944	p = 0.0242
	Interaction (CN X ESI)	F (1,20) = 9.390	p = 0.0061		F (1,20) = 5.616	p = 0.028
